# Modafinil Abrogates Methamphetamine-Induced Neuroinflammation and Apoptotic Effects in the Mouse Striatum

**DOI:** 10.1371/journal.pone.0046599

**Published:** 2012-10-04

**Authors:** Mariana Raineri, Betina Gonzalez, Belen Goitia, Edgar Garcia-Rill, Irina N. Krasnova, Jean Lud Cadet, Francisco J. Urbano, Veronica Bisagno

**Affiliations:** 1 Instituto de Investigaciones Farmacológicas (Universidad de Buenos Aires – Consejo Nacional de Investigaciones Científicas y Técnicas), Ciudad Autónoma de Buenos Aires, Buenos Aires, Argentina; 2 Laboratorio de Fisiología y Biología Molecular, Instituto de Fisiología, Biología Molecular y Neurociencias (Universidad de Buenos Aires – Consejo Nacional de Investigaciones Científicas y Técnicas), Ciudad Autónoma de Buenos Aires, Buenos Aires, Argentina; 3 Molecular Neuropsychiatry Research Branch, NIH/NIDA Intramural Research Program, Baltimore, Maryland, United States of America; 4 Center for Translational Neuroscience, Department of Neurobiology and Developmental Sciences, University of Arkansas for Medical Sciences, Little Rock, Arkansas, United States of America; The Scripps Research Institute, United States of America

## Abstract

Methamphetamine is a drug of abuse that can cause neurotoxic damage in humans and animals. Modafinil, a wake-promoting compound approved for the treatment of sleeping disorders, is being prescribed off label for the treatment of methamphetamine dependence. The aim of the present study was to investigate if modafinil could counteract methamphetamine-induced neuroinflammatory processes, which occur in conjunction with degeneration of dopaminergic terminals in the mouse striatum. We evaluated the effect of a toxic methamphetamine binge in female C57BL/6 mice (4×5 mg/kg, i.p., 2 h apart) and modafinil co-administration (2×90 mg/kg, i.p., 1 h before the first and fourth methamphetamine injections) on glial cells (microglia and astroglia). We also evaluated the striatal expression of the pro-apoptotic BAX and anti-apoptotic Bcl-2 proteins, which are known to mediate methamphetamine-induced apoptotic effects. Modafinil by itself did not cause reactive gliosis and counteracted methamphetamine-induced microglial and astroglial activation. Modafinil also counteracted the decrease in tyrosine hydroxylase and dopamine transporter levels and prevented methamphetamine-induced increases in the pro-apoptotic BAX and decreases in the anti-apoptotic Bcl-2 protein expression. Our results indicate that modafinil can interfere with methamphetamine actions and provide protection against dopamine toxicity, cell death, and neuroinflammation in the mouse striatum.

## Introduction

Methamphetamine (METH) is an illicit drug of abuse that can cause neuropsychiatric and neurotoxic damage in humans and animals. METH mechanisms of action include the reversal of the dopamine transporter (DAT), resulting in dopamine (DA) efflux from dopaminergic terminals [1]. METH also has pharmacological effects on a number of additional targets, including the vesicular monoamine transporter [2]. Neurotoxic effects of METH have been demonstrated in many species including rats, mice, guinea pigs, cats and monkeys (for a review, [2]). Animals, given repeated moderate to large doses of methamphetamine, experience significant loss of DA and serotonin (5-HT) in the striatum, cortex and in the olfactory bulb [3]. In addition, METH decreases striatal DAT binding as well as TH and DAT immunoreactivity [4], [5], [6]. Morphological studies suggest that reductions in DA markers and 5-HT system integrity are related to degeneration of DA and 5-HT axonal terminals [7]. Several studies have demonstrated that METH induces terminal deoxynucleotidyl transferase-mediated dUTP nick end labeling (TUNEL)-positive cells [8], [9]. In the striatum, the affected cell populations include gabaergic projection neurons and cholinergic and GABA-parvalbumin interneurons [9]. METH-induced cell death occurs by activation of mitochondria- and endoplasmic reticulum dependent cell death pathways [10].

In addition to these toxic effects, METH also causes reactive astrocytosis [11] and microglial activation [12] in the striatum. Basically, METH induces a substantial microglial response in the areas of the brain that show neuronal degeneration, and induces biochemical changes in microglia, that are similar to those that occur during neuroinflammation [12], [13]. These observations are consistent with the report that METH abusers exhibit elevated levels of peripheral benzodiazepine receptor binding, a marker for microglial activation [14].

Modafinil, a wake-promoting compound approved for the treatment of narcolepsy, is been prescribed “off label” for other psychiatric disorders such as attention-deficit-hyperactivity disorder and psychostimulant dependence [15]. Modafinil has been reported to affect a number of neurotransmitter systems including orexinergic, gabaergic and noradrenergic (for a review see [15]). In addition, modafinil was recently found to increase electrical coupling [16], [17]. Interestingly, modafinil also prevents the clearance of DA from the synaptic cleft by blocking DAT [18]. Modafinil also showed neuroprotective properties in animal models of Parkinson’s disease, such as reduced degeneration of dopaminergic neurons in the substantia nigra after partial transection of the nigrostriatal DA pathway and after injection of the neurotoxin MPTP [19], [20], [21], [22]. We have also shown that modafinil can block METH-induced DA depletion and decreased TH immunoreactivy in the striatum [23], but the exact mechanisms responsible for the neuroprotective actions of modafinil are unknown. Suggestions have been made about direct interference with processes that affect cell death such as energy metabolism, synthesis and release of neurotrophic factors, recovery of calcium homeostasis and DAT blocking properties [19], [20], [21], [23].

Since modafinil can protect against METH dopaminergic toxicity, one major aim of this study was to investigate if modafinil could also influence METH-induced astrocytosis and reactive microgliosis because these changes occur in conjunction with degeneration of DA terminals [12]. Here, we provide evidence that acute modafinil itself did not cause reactive gliosis but counteracted METH-induced microglial and astroglial activation in the striatum. Modafinil treatment also blocked METH-induced increases in the pro-apoptotic protein BAX, and decreases in the anti-apoptotic protein Bcl-2 in the mouse striatum.

## Materials and Methods

### Animals

Female C57BL/6 mice (20–25 g) from the School of Exact and Natural Sciences of the University of Buenos Aires (UBA) were housed in a light- and temperature-controlled room. Mice had free access to food and water. Principles of animal care were followed in accordance with “Guidelines for the Care and Use of Mammals in Neuroscience and Behavioral Research” (National Research Council, 2003) and approved by Universidad de Buenos Aires authorities (Protocol Number: A5801-01) using OLAW and ARENA directives (NIH, Bethesda, USA).

### Pharmacological Reagents

Drugs were purchased from either Sigma (St. Louis, MO) or Tocris (Ellisville, MO). Modafinil (racemic mixture of R- and S-enantiomers) was generously donated by Laboratorios Beta S.A. (Argentina).

### Pharmacological and Physiological Procedures

(+)-Methamphetamine hydrochloride (Sigma, St Louis, MO) was administered in a *binge* regimen: 4×5 mg/kg i.p. 2 h apart (calculated as free base). METH regimen used in this study was chosen according to studies done by Thomas et al. [12]. Modafinil (90 mg/kg, dissolved in DMSO-Arabic gum 5% in sterile saline solution) was injected twice, 60 min before the first and fourth METH injections, according to previous studies by Raineri et al. [23]. Control groups received the same volume of sterile saline and DMSO/saline, using the same schedule as METH and modafinil injections.

### Tissue Processing for Histochemical Studies

Animals were deeply anesthetized 48 hours or 6 days after the last METH injection with ketamine-xylazine (0.5 ml/kg i.p.; 1.4 ml/kg i.p., respectively) and then transcardially perfused with 30 ml of phosphate buffer saline (PBS) 10 mM (pH 7.4) followed by 80 ml of ice-cold paraformaldehide 4% (Sigma, USA) diluted in 0.1 M (pH 7.4) PB. Brains were dissected and placed in the same fixative solution for 16 hours at 4°C, and then the cerebral hemispheres were separated. One of the hemispheres remained immersed in PBS and until coronal vibratome sectioning (for lectin histochemical staining of microglia), while the other hemisphere remained in PBS/sucrose 30% for cryoprotection until coronal sections were cut throughout the striatum (for TH, DAT and GFAP immunostaining).

### Lectin Histochemical Staining of Microglia

Microglial staining was performed by staining fixed 50 µm coronal brain sections with HRP-conjugated isolectin B4 (ILB4) as described by Streit [24]. ILB4 stains microglia selectively in the central nervous system [12], and lectin binding is not detectable on astrocytes and oligodendrocytes [25]. Briefly, sections were floated 0.1 M PBS (pH 7.2) containing 3% H_2_O_2_ for 30 min, and washed 3 times in PBS 0.1% Triton X-100 for 10 min. Microglia were labeled with HRP-conjugated ILB4 (10 µg/ml in 0.1% Triton X-100) overnight at 4°C. Excess of ILB4 was removed by three washes with 0.1% PBS triton X-100 (5 min each) followed by a single wash in PBS before chromogenic reaction with 0.5 mg/ml 3,3′-diaminobenzidine (DAB) (Sigma, USA) and 0.015% H_2_O_2_. Sections were then rinsed, mounted on gelatin-coated slides, air-dried, dehydrated, cleared, and cover slipped. Brain sections from test drug-treated mice were processed simultaneously with controls and METH-treated mice to normalize staining among treatment groups.

**Figure 1:**
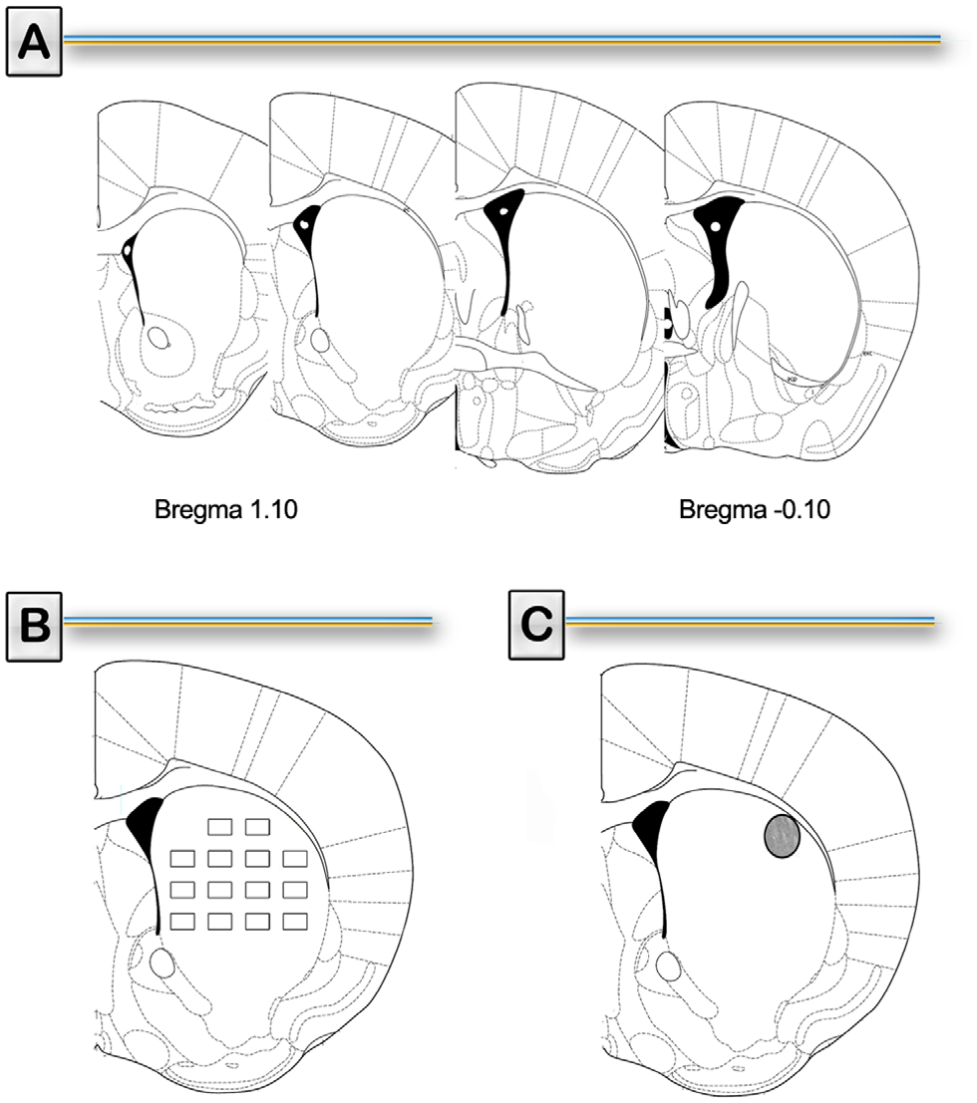
Representative diagrams used for data analysis. A) Representative coronal striatal sections (Bregma 1.10 to −0.10 mm) obtained from the Franklin and Paxinos atlas [45]. B) Representative coronal striatal section illustrating the placement of 14 probes (236 × 140 µm area) used for the evaluation of microglial and astroglial quantification (Mercator Pro; Explora Nova, France). C) Representative coronal striatal section containing the region of interest in the dorsolateral striatum from where TH and DAT immunoreactivity was determined.

Microglial cell count was performed as follows: light microscopic examination of vibratome sections revealed staining of microglial cells with ILB4-HRP throughout the striatum. Cells were classified according to their morphology as ramified, hyper ramified-reactive, or amoeboid-shaped microglia [26] from 4 independent sections from each treated mouse (Fig. 1A). All counts were performed by a person blind to treatment conditions. Ramified cells were considered resting microglia while cells expressing hyper ramified-reactive or amoeboid-shaped morphologies were considered activated microglia. Data were collected with the help of a stereology software (Mercator Pro; Explora Nova, France), coupled to a Nikon microscope. Cells were counted with a semiautomatic system that allows tagging the position of the counted cells within 14 probes (236×140 µm area of the striatum) randomly selected by the Mercator software in the outlined striatum (Fig. 1B).

### Immunohistochemistry for TH, DAT and GFAP

Immunostaining for TH and GFAP were performed on 25 µm free-floating coronal serial slices from striatal sections (Fig. 1A). During all staining procedures 0.1 M PBS, 0.15% Triton X-100 (PBS-T) was used for diluting all immunoreagents and for washing after incubating with antibodies. Sections were incubated in 0.6% H_2_O_2_ (3% in the case of GFAP immunoassay) followed by 2% normal goat serum (NGS) in PBS-T and exposed to a rabbit anti-TH antiserum (1∶1000, Pel Freez Biologicals, USA), rabbit anti-GFAP antibody (1∶500, Sigma, USA) overnight at 48 C. After washing, sections were incubated for 2 h in biotinylated goat antirabbit IgG antiserum (1∶500, Sigma, USA) followed by the avidin-biotin peroxidase complex (1∶125, Vectastain, ELITE ABC kit, Vector Laboratories). Chromogenic reactions were induced with 0.5 mg/ml DAB, and 0.015% H_2_O_2_. Sections were then rinsed, mounted on gelatin-coated slides, air-dried, dehydrated, cleared, and coverslipped. The same protocol was applied for DAT immunostaining using DAT monoclonal primary antibody (1∶500, Millipore, USA) and HRP-conjugated anti-rat antibody (1∶1000, Sigma, USA).

GFAP-immunoreactive area was quantified as follows: light microscopic examination of microtome sections revealed GFAP-positive staining throughout the striatum. Data were collected with the help of mapping software (Mercator Pro; Explora Nova, France), coupled to a Nikon microscope. The immunoreactive area was determined with a semiautomatic system that allows tagging the percent of stained area within 14 probes (236×140 µm area of the striatum) randomly selected by the Mercator software in the outlined striatum (Fig. 1B). Percent of stained area was calculated generating an average count for each treated subject. In order to differentiate specific staining from background, a color threshold was used in the process. Quantification was performed on 4 slices per animal (Fig. 1A) by a person blind to treatments.

TH and DAT levels in the striatum were quantified as follows: histological sections stained with anti-TH and anti-DAT antibodies were observed under a Nikon light microscope with a CCD camera for image acquisition. For measurement of integrated density, slice images were obtained with a 10X objective under standard conditions (magnification, brightness). Unequal brightness distribution (shading) was corrected using the *a posteriori* shading correction plug-in (NIH ImageJ). A threshold was applied to all images according to the average of the automatic values of threshold obtained for the saline treated animals in order to avoid non-specific staining. Integrated density was then measured in the dorsolateral striatum from striatal TH or DAT from 4 stained sections per animal with an ellipsoidal region of interest (613,400 µm2, Fig. 1C) using ImageJ software.

### Western Blot

Mice were sacrificed at 16 h after drug treatment. Brains were quickly removed and the striata were dissected out and stored at –70°C for Western blot analyses. Tissue homogenates were prepared in a solution containing 50 mM Tris-HCl pH 7.5, 150 mM NaCl, 0.1% Triton X100, 0.5% sodium deoxycholate, 0.1% SDS, 1 mM PMSF, 5 µg/ml leupeptin, and 5 µg/ml aprotinin. After removal of cell debris by centrifugation, the protein concentration of the cell lysate was determined. The homogenates were combined with loading buffer containing 4% SDS, 20% glycerol, 10% β-mercaptoethanol, 125 mM Tris, (pH 6.8) and boiled at 100 °C for 5 min. Protein samples (50–100 µg) were separated by 12.5% SDS-PAGE, and the separated proteins transferred to a PVDF membrane. Immunoblotting was performed using a polyclonal rabbit antibody to BAX (1∶500, Santa Cruz Biotechnology), or to Bcl-2 (1∶500, R&D Systems, MN, USA). Immune complexes were detected with anti-rabbit secondary antibodies and chemiluminescence reagents (Amersham, NJ, USA). Then membranes were stripped and reprobed with monoclonal antibody against α-tubulin (1∶3000, Sigma, USA) to confirm equal loading and transfer of samples.

### Core Body Temperature Measurement

Core body temperature was recorded with a Bat-lO thermometer coupled to a RET-3 mouse rectal probe (Physitemp, Inc., NJ, USA) lubricated with mineral oil. Mice temperatures were recorded 1 h before the first modafinil injection and at different time intervals thereafter (up to 16 hours after the last METH injection).

### Statistical Analysis

Sigmaplot 10.0 (Systat Software, CA) and InfoStat software (www.infostat.com.ar) were used for statistical comparisons. Statistics were performed using either Kruskal Wallis one-way ANOVA on Ranks (for TH and DAT immunoreactivity), or one-way ANOVA followed by Tukey’s (glia immunoreactivity), or Fisher’s LSD (apoptotic protein expression) *post hoc* tests. In the case of core temperature repeated measures two-way ANOVA followed by Fisher’s LSD was performed. Differences were considered significant if p<0.05.

## Results

### Modafinil Treatment Prevents METH-induced Decrease in TH Immunoreactivity in the Striatum

**Figure 2:**
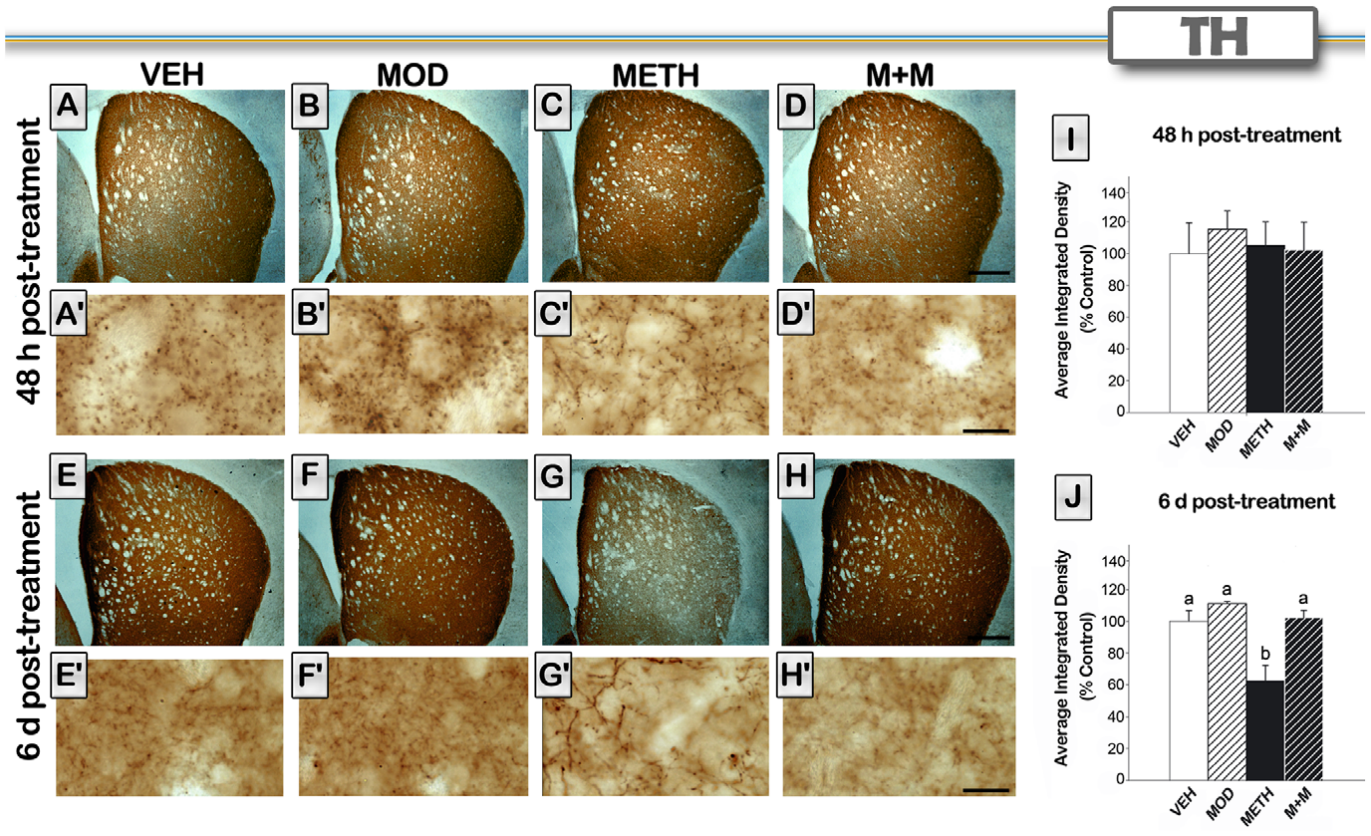
Modafinil treatment prevents METH-induced decrease in TH immunoreactivity in the striatum. Representative images of the striatum from brains of animals treated with either VEH (A, E), MOD (B, F), METH (C, G) M+M (D, H), scale bar, 500 µm, and their respective higher magnification photomicrographs (A′–H′), scale bar, 10 µm. Brain sections of animals sacrificed 48 hours (A–D) or 6 days (E–H) after the last METH injection were processed for TH immunoreactivity. Note the decrease in intensity and the changes in TH fiber morphology in the METH treated group 6 days after treatment. Average integrated density (% control) was determined in a region of interest in the dorsolateral striatum in mice (n = 4−8) sacrificed 48 hours (I) or 6 days (J) after the last METH injection. Values are expressed as mean ± SEM. Kruskal Wallis ANOVA on ranks, different letters: p<0.05.

Several reports have indicated that repetitive injections of METH decrease TH immunoreactivity in striatal areas [27]. Because the striatal pattern of dopamine damage caused by METH is more profound in lateral areas [12], we decided to assess the integrity of dopaminergic terminals by examining TH-immunoreactive fibers in the dorsolateral region of the striatum. Representative photomicrographs and quantification of TH staining in the striatal sections are shown in Figure 2. 48 h after METH treatment there was no significant change on TH immunostaining [H = 1.15, p = 0.7659]. We did observe a decrease 6 days after treatment [H = 13.50, p<0.01] in the METH group on TH levels. Also, a change in the morphology of TH-positive fibers was observed only in the METH group (Fig. 2 A–H). The TH integrated density in the METH group was significantly decreased compared to that of the VEH group (p<0.05). Modafinil co-administration was able to prevent the METH-induced decrease in TH immunoreactivity levels (p<0.01). Neither modafinil by itself (MOD) nor the co-administration of modafinil and METH (M+M) altered TH levels compared to vehicle-treated mice (VEH) at any time point.

### Modafinil Treatment Prevents METH-induced Decrease in DAT Immunoreactivity in the Striatum

**Figure 3:**
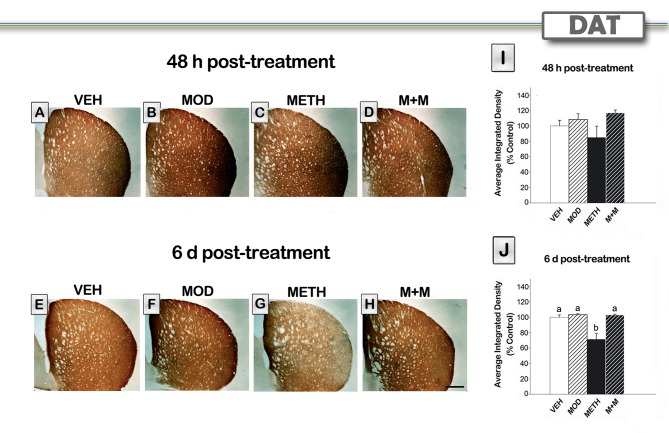
Modafinil treatment prevents METH-induced decrease in DAT immunoreactivity in the striatum. Representative images of the striatum from brains of animals treated with either VEH (A, E), MOD (B, F), METH (C, G), or M+M (D, H), scale bar, 500 µm. Brain sections of animals sacrificed 48 hours (A–D) or 6 days (E–H) after the last METH injection were processed for DAT immunoreactivity. Note the decrease in intensity observed in the METH-treated group 6 days after treatment. Average integrated density (% control) was determined in a region of interest in the dorsolateral striatum in mice (n = 5−8 sacrificed 48 hours (I) or 6 days (J) after the last METH injection. Values are expressed as mean ± SEM. Kruskal Wallis ANOVA on ranks, different letters: p<0.05.

We also investigated the effects of METH on DAT immunoreactivity. Representative photomicrographs of DAT staining in the striatum of each group are shown in Figure 3. The METH-treated group showed a decrease in DAT immunoreactivity at 6 days after treatment, but not after 48 h. Neither modafinil by itself nor the co-administration of modafinil and METH altered DAT levels compared to the VEH group. After 48 hours, no effect on DAT immunoreactivity was observed [H = 5,93, p = 0.1153], as shown in Figure 3. In contrast, 6 days after treatment we found significant treatment effects [H = 10,42, p<0.05]. *Post hoc* test showed a decrease in DAT density in METH-treated animals compared to VEH values (p<0.05). Modafinil pretreatment protected against METH-induced DAT decrease (p<0.01).

### Modafinil Treatment Prevents METH-induced Striatal Microglial Activation

**Figure 4:**
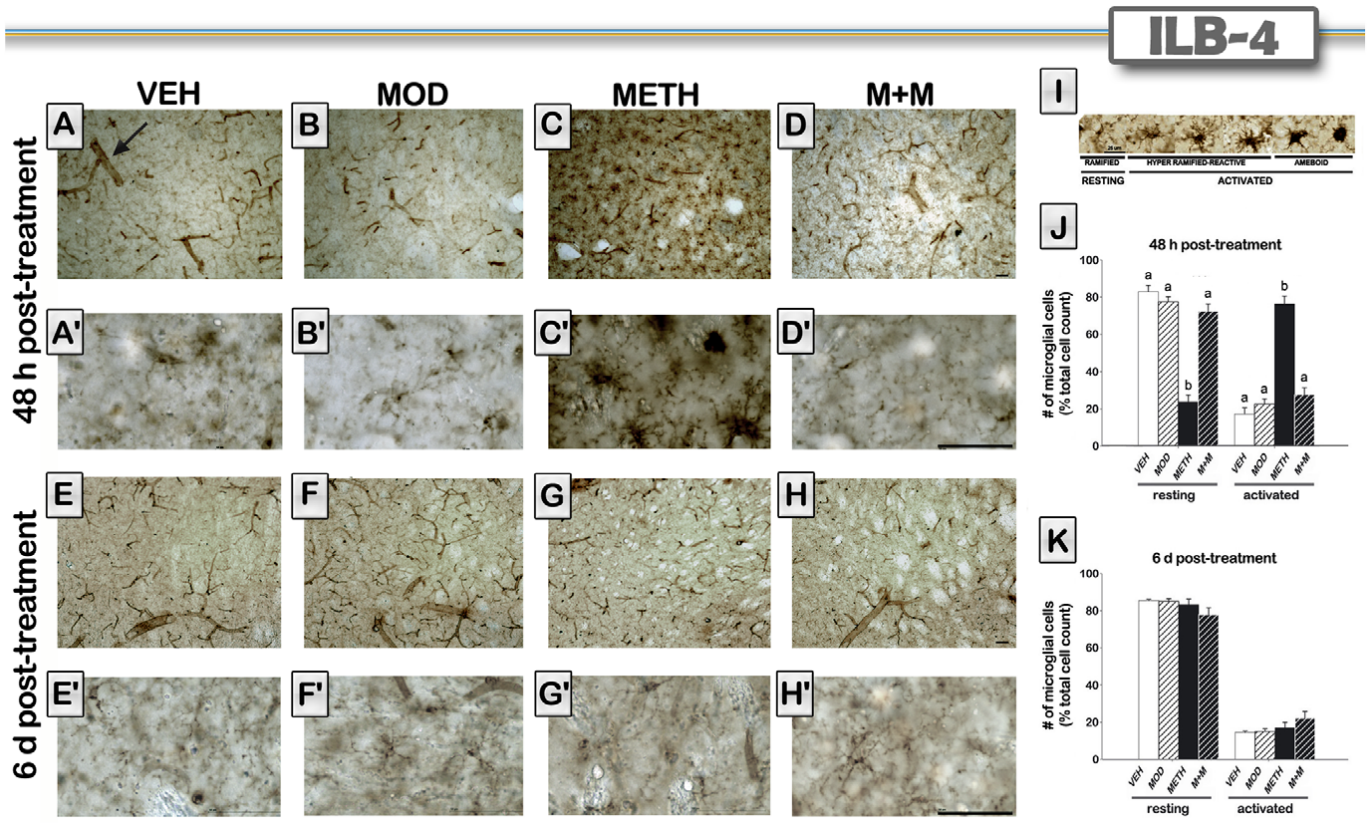
Modafinil treatment prevents METH-induced striatal microglial activation. Representative images of the striatum from brains of animals treated with either VEH (A, E), modafinil (MOD) (B, F), METH (C, G), or M+M (D, H), and the respective higher magnification photomicrographs (A′–H′). Brain sections of animals sacrificed 48 hours (A–D) or 6 days (E–H) after the last METH injection were labeled with ILB4 isolectin. Note the change in microglia cell morphology towards a more activated phenotype in the METH treated group 48 h after treatment, and that blood vessel were also labeled with ILB4 lectin (see arrow in panel A). Representative images of microglia showing the different morphologies observed in resting and activated states (I). Microglia averaged cell count at their resting or activated states obtained from mice (n = 4−8) sacrificed 48 hours (J) or 6 days (K) after the last METH injection. Values are expressed as mean ± SEM. One-way ANOVA followed by Tukey, different letters: p<0.001. Scale bars, 50 µm.

The neurotoxic regimen of METH used in the present study caused a substantial increase in microglial staining. Because METH-induced microglial activation increases maximally at 24 and 48 h after a METH binge [12], we decided to include an early end point for microglial activation (48 hours). Also, 6 days after METH, microglia was quantified because it has been reported that microglial cells return to control levels after 7 days post METH treatment [12]. Representative sections from all treatment groups are shown in Figure 4. Resting and activated microglial morphologies are also shown in Fig. 4I. Resting microglia displayed thin branching processes that extended radially from small oblong somata, whereas activated microglia exhibited marked hyperplastic changes that included increases in soma size, presence of thicker, branched processes, and an increase in staining intensity, as previously described by LaVoie et al. [26].

As expected, 48 h after treatment, METH induced a clear increase in hyperramified-reactive and amoeboid cells (activated states), and a decrease in the percentage of ramified (resting) microglial cells. Modafinil itself did not alter the activation pattern of microglia, and the modafinil and METH (M+M) group showed similar levels of microglia staining indistinguishable from the VEH-treated group (Fig. 4J). ANOVA showed significant differences among treatment groups [F(3,14) = 50.50, p<0.0001]. METH increased activated microglia as indicated by *post hoc* tests (p<0.001), while the other groups showed no significant differences when compared to VEH-treated mice. In addition, we quantified amoeboid-shaped glial cells (Table 1), as they are considered to be the final step in microglial activation. We found significant differences among groups [H = 9.15; p<0.01], with only METH-treated animals showing an increase in amoeboid cell number (p<0.05). No differences were observed among groups 6 days after METH treatment (Fig. 4K). Also, amoeboid-shaped cells (Table 1) were not observed in any group at this time point.

**Table 1:** Modafinil treatment prevents METH-induced striatal amoeboid-shaped microglial differentiation.

Treatment	Time after treatment	
	48 h	6 d
VEH	0	0
MOD	0.25±0.25	0
METH	14.25±8.17*	0.28±0.18
M+M	0	0

ILB4-positive cell number per mice with amoeboid-shaped morphology was estimated.

Values are expressed as mean number of amoeboid microglia ± SEM (n = 4−8). Kruskal Wallis ANOVA on ranks was performed to draw comparisons between the different groups: *p<0.05.

### Modafinil Treatment Counteracts METH-induced Striatal Astroglial Activation

**Figure 5:**
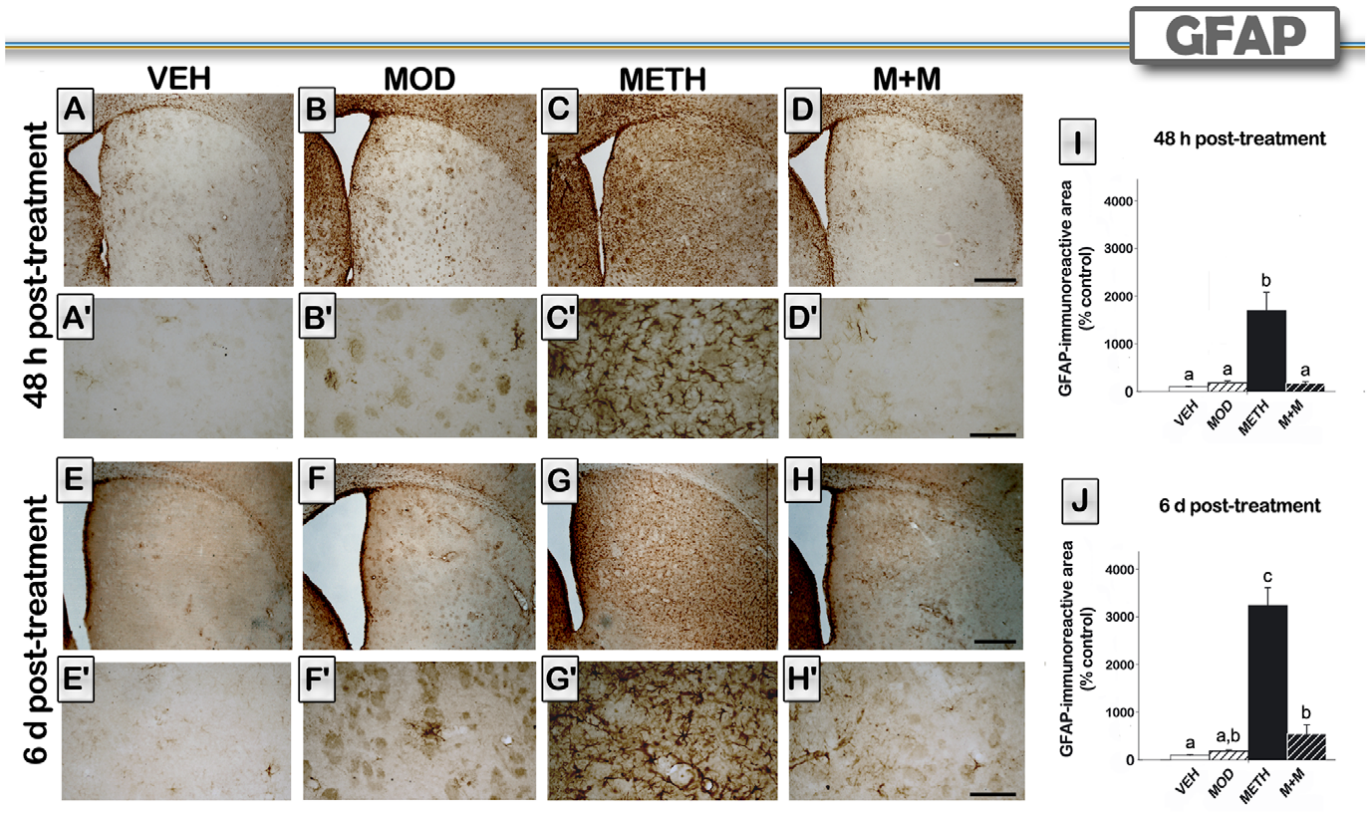
Modafinil treatment counteracts METH-induced striatal astroglial activation. Representative images of the striatum from brains of animals treated with either VEH (A, E), MOD (B, F), METH (C, G), or M+M (D, H), scale bar, 500 µm, and their respective higher magnification photomicrographs (A′–H′), scale bar, 100 µm. Brain sections of animals sacrificed 48 hours (A–D) or 6 days (E–H) after the last METH injection were processed for GFAP immunoreactivity. Percentage of immunoreactive area was determined in mice (n = 5−8) sacrificed 48 hours (I) or 6 days (J) after the last METH injection. Values are expressed as mean ± SEM. One-way ANOVA followed by Tukey, different letters: p<0.01.

GFAP is a standard marker of astrogliosis [28]. METH administration is known to increase GFAP immunoreactivity in the striatum of mice [29]. Astrogliosis was evaluated by immunohistochemistry in the striatum of mice at both 48 hours and 6 days post METH. Representative sections from all treatment groups are shown in Figure 5. A clear increase in GFAP-immunoreactivity was observed in METH-treated groups at 48 h [F(3,19) = 30,11, p<0.0001] and 6 days [F(3,23) = 50,30, p<0.0001] after treatment. At 48 h post treatment, modafinil completely blocked METH-induced astroglial activation (p<0.001) but this effect was only partially observed after 6 days (p<0.01). The M+M group showed an increase in immunoreactive area when compared with VEH values only 6 days after treatment, but the increase in immunoreactive area was significantly lower than the one observed for the METH group (p<0.001). Modafinil by itself did not induce astroglial activation at any time.

### Modafinil Co-administration Prevents METH-induced Increase of Pro-apoptotic, and Decrease of Anti-apoptotic, Markers in the Striatum

**Figure 6:**
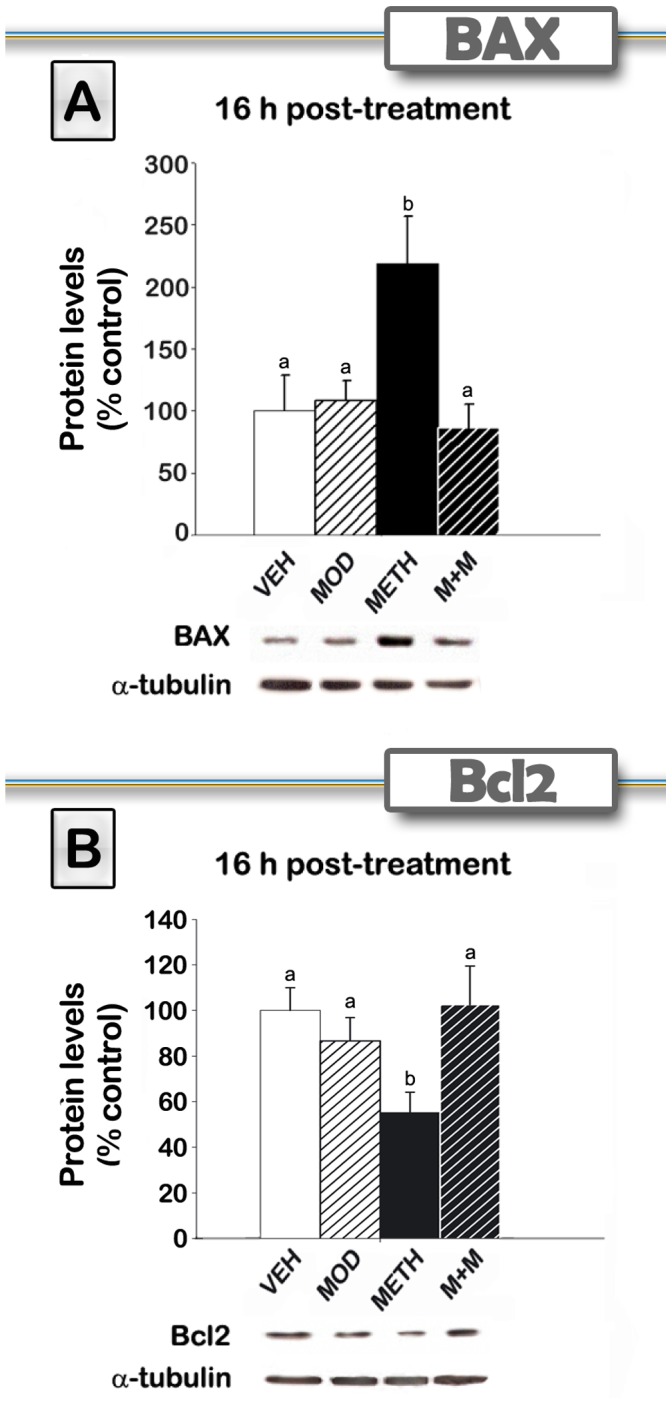
Modafinil treatment prevents METH-induced increase of pro-apoptotic and decrease of anti-apoptotic markers in the striatum. Expression of pro-apoptotic BAX (A) and anti-apoptotic Bcl2 (B) proteins in the striatum, measured by Western blot analyses. Striata were obtained 16 hours after VEH, MOD, METH or M+M treatments (n = 5−9).Values represent mean ± SEM (% of control). One-way ANOVA followed by Fisher’s LSD. different letters: p<0.05.

METH is known to alter the expression of proteins that participate in apoptotic pathways in the brain [2]. At 16 hours post METH, these proteins were found to be modified by METH [10], [30]. In order to investigate the effects of modafinil pretreatment on METH-induced cell apoptosis, we measured striatal BAX and Bcl-2 protein expression by Western blot in the striatum of mice treated and euthanized 16 hours after treatment (Fig. 6). METH-treated group displayed an increase in BAX expression [F(3,21) = 4,96, p<0.01], together with a decrease in Bcl-2 expression levels [F(3,27) = 3,58, p<0,05]. Modafinil pretreatment was able to counteract these effects and modafinil by itself did not modify the expression of these two proteins. METH treatment increased BAX (p<0.01) and lowered Bcl-2 expression (p<0.05), while modafinil co-administration prevented these changes. Modafinil alone did not display significant differences relative to VEH levels.

### Modafinil Treatment Modulates METH-induced Hyperthermia

**Figure 7:**
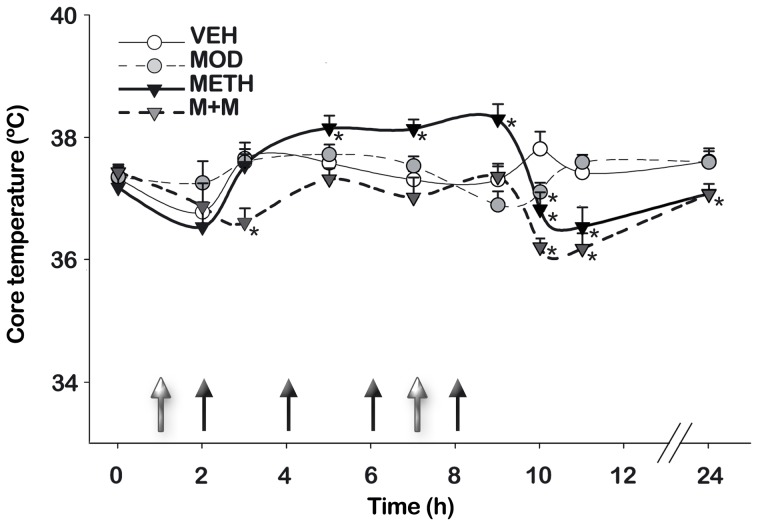
Modafinil treatment modulates METH-induced hyperthermia. Mice body temperatures were recorded 1 h before the first MOD injection and at different intervals thereafter. Black filled arrows indicate the time of METH injection while grey filled arrows indicate the time of MOD injection. Data is presented as mean core temperature (°C) at the indicated times ± SEM from animals treated with VEH (white circle), MOD (grey circle, dotted line), METH (Black inverted triangle), or M+M (dark grey inverted triangle, dotted line). Repeated measures two-way ANOVA followed by Fisher’s LSD, *p<0.05 vs. VEH.

A link between METH-induced hyperthermia and dopaminergic neurotoxicity in mice has been suggested [31]. Therefore, we sought to determine whether modafinil pretreatment could influence METH-induced hyperthermia. Core body temperature was measured 1 h before the first modafinil injection and at different time intervals thereafter (Figure 7). Two-way ANOVA showed that treatments and time produced a significant change [F(treatment) (3,130) = 19,63, p<0.0001, F(time) (5,130) = 19,63, p<0.0001], and that there was an interaction between the two factors [F(treatment × time) (15,130) = 4.73, p<0.0001]. *Post hoc* tests indicated that i) METH-treated mice showed an hyperthermic response, which was significantly different from VEH group at the second, third, and last METH injections (p<0.05), ii) modafinil administration alone had no effect on body temperature at any time, and iii) modafinil co-administration with METH (M+M group) significantly inhibited METH-induced hyperthermia (p<0.05). Two hours after the last METH injection a hypothermic effect was observed in METH and in M+M treated groups.

## Discussion

The main finding of this study is that modafinil treatment was able to block different METH-induced toxic effects in the mouse striatum. These protective effects included: 1) prevention of METH-induced decrease of TH and DAT immunostaining, 2) blocking of METH-induced increase in the pro-apoptotic protein BAX, and decrease in the anti-apoptotic Bcl-2 protein, 3) prevention of METH-induced microglial and astroglial activation, as well as 4) inhibition of METH-induced hyperthermia.

METH toxicity can be manipulated at various levels, based on its mechanisms of actions. METH enters DA neurons through the DAT and via passive diffusion. In the cell, it accumulates in vesicles, disrupts the pH gradient required for vesicular DA sequestration, and displaces DA into the cytoplasm [32]. DA accumulates in the cytoplasm, which alters the concentration gradient and likely serves to favor the reverse transport of DA via the DAT [33]. DA is an important component of the mechanisms that underlie METH neurotoxicity (see [2]). METH-induced cell death is related to intraterminal DA autoxidation and generation of reactive oxygen species (hydrogen peroxide), quinones, and semiquinones, with subsequent induction of neuronal apoptosis [2]. METH-induced alterations in the activity of monoamine transporter proteins have also been reported to contribute to persistent dopaminergic deficits [34]. Although several mechanisms have been suggested for the neurochemical effects of modafinil [15], its neuroprotective effects against METH toxicity might be more related to its actions as a DAT blocker [18] Modafinil is thought to interact with DAT at a different site than cocaine [35], [36]. Therefore, one suitable explanation for modafinil effects against METH striatal inflammation might be related to modafiniĺs DAT- blocking properties. Modafinil acting as a DAT blocker may have decreased the entry of METH into dopaminergic terminals, avoiding the toxic consequences of high levels of DA into theterminals and the synaptic cleft. This suggestion is compatible with the fact that DAT knockout mice are protected against METH-induced DA depletion, reactive astrocytosis, and reactive oxygen species production in the striatum [37]. This idea is also consistent with the observation that administration of the DAT inhibitor, methylphenidate, 1 hour after METH treatment can also block METH-induced toxicity [38].

METH can cause neuronal apoptosis in addition to terminal degeneration [5], [9], [2]. Some studies have demonstrated that METH can cause neuronal apoptosis in the cortex and striatum of rodents [10], [39], [30] by increasing caspase activity and the expression of proapoptotic Bcl-2 family related proteins, including BAD and BAX genes [2]. In the present study, we show that METH-treated mice displayed an increase in the pro-apoptotic marker BAX together with a decrease in the anti-apoptotic marker Bcl-2. The present findings of METH-induced decreases in the anti-apoptotic protein Bcl-2, but increases in the pro-apoptotic proteins BAX, are consistent with those observed after single large doses of METH [10]. We found, for the first time, that modafinil pretreatment was able to counteract these effects. Mitochondrial dysfunctions have been reported to influence METH toxicity [2]. Under the toxic METH regimen used in the present study, a clear increase in hyperramified-reactive and amoeboid cells (activated states), and a decrease in the percentage of ramified (resting state) microglial cells, were observed. These effects are consistent with a previous report by Thomas et al. [12], who showed (using the same METH regimen on C57BL/6 female mice) that microglial staining was increased maximally at 24 and at 48 hours after METH. Microglial counts returned to control levels after 7 days post METH. As expected, a clear increase in GFAP-immunoreactivity was also observed in METH-treated group at 48 h and 6 days after METH treatment. Modafinil by itself did not induce microglial or astroglial activation at any time point. The fact that modafinil did show preventive effects on glial cells suggests that modafinil blocked early toxic events induced by a METH binge in striatal tissue.

A major clinical danger associated with high-dose METH abuse in humans is the development of hyperthermia [2]. Therefore, we were interested in evaluating whether modafinil pretreatment might modulate METH-induced hyperthermia. We found, after the second, third, and last METH injections, a hyperthermic response in the METH-treated group that was not present in the M+M group, indicating that modafinil co-administration prevented METH-dependent increases in body temperature. Modafinil by itself did not alter core body temperature. Hypothermia was observed at 2 h after the last METH injection in both METH and M+M-treated animals. These effects are consistent with previous reports where the hyperthermic response of METH was followed by a hypothermic response, probably due to altered homeostatic mechanisms induced by the binge METH injections [13]. The neurotoxic effects of METH can be modulated by drugs or treatments that prevent hyperthermia [11], [31], making it difficult to determine whether hyperthermia is a direct participant in drug-induced neurotoxicity or a response that is coincident but does not contribute to nerve terminal damage [13]. Also, it has been previously shown that increases in temperature lead to increases in DAT function [40], therefore, it is possible that, at least in part, increased core temperature enhances METH-induced DA neurotoxicity by amplifying a DAT-dependent neurotoxic cascade [41]. Some authors have also suggested that core temperature responses to METH (i.e., hypothermia or hyperthermia) cannot predict the subsequent neurochemical response (i.e., toxicity or neuroprotection) to drug administration, and that there is a suitable scenario where hyperthermia is neither necessary nor sufficient for amphetamine-induced neurotoxicity [13]. It needs to be pointed out that, even though increases in core temperature are not essential for the expression of METH-induced DA neurotoxicity (either in rodents or primates), the possibility still exists that hyperthermia might contribute to some extent to METH-induced toxicity. Therefore, the fact that modafinil pretreatment prevented METH- induced hyperthermia may contribute to its neuroprotective actions. Considerable evidence has accumulated indicating that stimulation of central DA receptors can produce thermoregulatory responses in most species [42]. The evidence points to the involvement of tubero-infundibular neurons of the pre-optic hypothalamus in temperature regulation, although the mesolimbic and nigrostriatal pathways have also been implicated [43]. In any case, increased release of DA in any of these regions might, in part, be responsible for METH-induced thermoregulatory dysfunctions. Therefore, modafinil might have prevented METH-induced hyperthermia via the same mechanisms described earlier.

Altogether, our results indicate that modafinil treatment can provide protection against DA toxicity, cell death, and neuroinflammation. Modafinil-mediated neuroprotection might be related to DAT antagonism, since other DAT inhibitors also block METH-induced toxicity [38]. Thus, by blocking one of the sites that METH uses to enter dopaminergic terminals, modafinil might have interfered with a wide range of toxic processes induced by METH. This suggestion is consistent with the idea that inhibition of DAT might be a critical factor mediating the efficacy of modafinil in treating cocaine and METH dependence [44], [18]. However, we cannot rule out that modafinil neuroprotective effects against METH toxicity might be mediated by an indirect mechanism linked to thermoregulatory processes. Modafinil, by decreasing the hyperthermia induced by METH throughout the binge, may have decreased METH strength to induce striatal toxic effects. Further mechanistic studies are needed to elucidate which factors contribute to modafinil neuroprotection against METH toxicity, and thermoregulatory effects induced by modafinil certainly need to be explored. GABAergic neurotransmission, dopamine and their interactive effects might also be good candidates for further research. Indeed, it has been shown that modafinil causes a dose dependent decrease of GABA in rat cortex, hypothalamus, thalamus, substantia nigra, hippocampus and also in dorsal and ventral striatum (reviewed in [46]). These effects on extracellular GABA seem to be mediated by modafinil on other neurotransmitter systems, i.e. dopaminergic and serotonergic [46]. The striatum is a complex neuronal network composed by more than 90% of GABAergic cells that receive massive dopamine input and that are responsive to dopamine toxicity [2]. Effects on GABAergic neurotransmission mediated by modafinil (in different brain areas) might in turn contribute to its neuroprotective profile against METH toxicity.

Recent clinical data have supported the use of modafinil for treatment of chronic METH addiction and relapse prevention [47], [48]. Also preclinical studies [49] have found that modafinil could block METH-primed reinstatement to METH seeking in a model of relapse in both male and female rats. In a related study, Reichel and See [50] found that chronic/prophylactic treatment with modafinil during withdrawal also attenuated subsequent relapse to METH seeking in rats. Finally, our results suggest that, when used as a treatment for METH dependence, modafinil may also prevent METH toxic and inflammatory consequences in the human brain.

## Acknowledgments

Dr. Bisagno has been authorized to study drug abuse substances in animal models by A.N.M.A.T. (National Board of Medicine Food and Medical Technology, Ministerio de Salud, Argentina). Dr. Betina Gonzalez is a recipient of a Postdoctoral Award from Fundación Bunge y Born.
